# Antibacterial Activities of Wasabi against *Escherichia coli* O157:H7 and *Staphylococcus aureus*

**DOI:** 10.3389/fmicb.2016.01403

**Published:** 2016-09-21

**Authors:** Zhongjing Lu, Christopher R. Dockery, Michael Crosby, Katherine Chavarria, Brett Patterson, Matthew Giedd

**Affiliations:** ^1^Department of Molecular and Cellular Biology, Kennesaw State University, KennesawGA, USA; ^2^Department of Chemistry and Biochemistry, Kennesaw State University, KennesawGA, USA

**Keywords:** wasabi, allyl isothiocyanate, *E. coli* O157:H7, *S. aureus*, foodborne pathogen

## Abstract

*Escherichia coli* O157:H7 and *Staphylococcus aureus* are two of the major pathogens frequently involved in foodborne outbreaks. Control of these pathogens in foods is essential to food safety. It is of great interest in the use of natural antimicrobial compounds present in edible plants to control foodborne pathogens as consumers prefer more natural “green” foods. Allyl isothiocyanate (AITC) is an antimicrobial compound naturally present in wasabi (Japanese horseradish) and several other edible plants. Although the antibacterial effects of pure AITC and wasabi extract (essential oil) against several bacteria have been reported, the antibacterial property of natural wasabi has not been well studied. This study investigated the antibacterial activities of wasabi as well as AITC against *E*. *coli* O157:H7 and *S*. *aureus*. Chemical analysis showed that AITC is the major isothiocyanate in wasabi. The AITC concentration in the wasabi powder used in this study was 5.91 ± 0.59 mg/g. The minimum inhibitory concentration (MIC) of wasabi against *E. coli* O157:H7 or *S. aureus* was 1% (or 10 mg/ml). Wasabi at 4% displayed higher bactericidal activity against *S. aureus* than against *E. coli* O157:H7. The MIC of AITC against either pathogen was between 10 and 100 μg/ml. AITC at 500 μg/ml was bactericidal against both pathogens while AITC at 1000 μg/ml eliminated *E. coli* O157:H7 much faster than *S. aureus*. The results from this study showed that wasabi has strong antibacterial property and has high potential to effectively control *E. coli* O157:H7 and *S. aureus* in foods. The antibacterial property along with its natural green color, unique flavor, and advantage to safeguard foods at the point of ingestion makes wasabi a promising natural edible antibacterial plant. The results from this study may be of significant interest to the food industry as they develop new and safe foods. These results may also stimulate more research to evaluate the antibacterial effect of wasabi against other foodborne pathogens and to explore other edible plants for their antimicrobial properties. To our knowledge, this is the first report on the antibacterial activity of wasabi in its natural form of consumption against *E. coli* O157:H7 and *S. aureus*.

## Introduction

Foodborne pathogens continue to be a serious threat to public health worldwide. Many multi-state foodborne outbreaks have been linked to a variety of ready-to-eat foods such as caramel apples, salads, pre-packaged leafy greens, sushi made with frozen raw tuna, cheeses, pizza, bologna, nut butter, and ice cream as well as frozen yogurt (CDC, 2016). These ready-to-eat foods were either improperly prepared (insufficient washing or cooking) or improperly refrigerated, or contaminated after processing by food handlers with poor personal hygiene. *E. coli* O157:H7 and *S. aureus* are two major foodborne bacterial pathogens frequently involved in these foodborne outbreaks. Control of these two pathogens as well as other pathogens in foods, especially ready-to-eat foods at the point of consumption, is essential to food safety.

Many physical and chemical methods have been used to control foodborne pathogens such as pasteurization, cooking, drying, radiation, acidification, salting, or addition of preservatives. However, as more consumers prefer fresh, less processed, more natural or “green” foods, it is of great interest to search natural antimicrobial agents present in edible plants to control pathogens in foods, especially in ready-to-eat foods which are not reheated before eating – an important step that would kill most bacterial pathogens, if they were in the food. It has been known that certain herbs, spices, plant extracts, and essential oils have antibacterial activities which come from certain phytochemicals present in the plants (Cowan, 1999; Nascimento et al., 2000; Aboaba et al., 2006; Shahid et al., 2009; Vashist and Jindal, 2012; Vinoth et al., 2012; Gobalakrishnan et al., 2013). Phytochemicals are a wide variety of chemicals naturally present in plants. The well-known phytochemicals include carotenoids, flavonoids, vitamins C and E, and folic acid. Thousands of phytochemicals have been identified, but only a small fraction has been closely studied (Vashist and Jindal, 2012). Certain phytochemicals are responsible for color and other organoleptic properties of the plants. Although not established as essential nutrients, phytochemicals may have biological significance benefiting human health. Many phytochemicals show antioxidant or anti-cancer activities (Zhang et al., 2003; Singh et al., 2005) and others show antibacterial potential (Rios and Recio, 2005; Arora and Kaur, 2007; Kaur and Arora, 2009). Although certain phytochemicals are available as dietary supplements, potential health benefits of phytochemicals may best be derived from consumption of whole foods.

Wasabi (*Wasabia japonica*, Japanese horseradish) is an edible plant containing a variety of phytochemicals. Wasabi belongs to the *Brassicaceae* family, which also includes horseradish, radish, mustard, and cabbage. Wasabi grows naturally along stream beds in mountain river valleys in Japan. Today, wasabi also grows in parts of China, Korea, New Zealand, and North America where it is cool, shady, and humid. Wasabi has an extremely strong pungency. Its hotness is more akin to that of a hot mustard than that of the capsaicin in a chili pepper, producing vapors that stimulate the nasal passages more than the tongue. Wasabi has been traditionally consumed as a spice and a condiment with sushi and a variety of other foods for centuries in Japan. More recently, wasabi has found widespread appeal in western cuisine owing to its unique hot taste, pungent smell, and bright green color (Sultana et al., 2002). It is also believed that wasabi has powerful herbal medicinal action, contributing to the safety of eating raw fish and other foods (Shin et al., 2004; Yano et al., 2006). A number of studies reported that wasabi has antibacterial property against foodborne pathogens such as *E*. *coli*, *Salmonella typhimurium*, *Pseudomonas aeruginosa*, *S. aureus*, and *Helicobacter pylori* (Nishida, 1958; Inoue et al., 1983; Kanemaru and Miyamoto, 1990; Shin et al., 2004). However, the antimicrobial activity of wasabi has not been well studied. Most researches used wasabi extract (less natural than wasabi) in the study of the antimicrobial property of wasabi. So far, there is only one report about the antimicrobial activity of wasabi, which is against *Vibrio parahaemolyticus* (Hasegawa et al., 1999). There is no study of the antibacterial activity of wasabi against other major foodborne pathogens including *E*. *coli* O157:H7 and *S*. *aureus*. As more consumers use wasabi in their foods, especially ready-to-eat foods (at the point of consumption), antimicrobial activity data directly from wasabi would be far more valuable because they are closer to reality. Although many studies showed that isothiocyanates (ITCs), mainly allyl isothiocyanate (AITC), are responsible for the antibacterial activity as well as the pungency in wasabi (Foter and Golick, 1938; Forter, 1940; Isshiki et al., 1992; Masuda et al., 1996; Kyung and Fleming, 1997; Lin et al., 2000a,b; Shin et al., 2004; Luciano and Holley, 2009; Dufour et al., 2012), very few studies have compared the antibacterial activity of AITC with that of wasabi against foodborne pathogens such as *E*. *coli* O157:H7 and *S*. *aureus*.

The aims of this study were to explore the antibacterial potential of wasabi against *E*. *coli* O157:H7 and *S*. *aureus*, specifically, to determine the minimum inhibitory concentrations (MICs) of wasabi against *E*. *coli* O157:H7 and *S*. *aureus*, and to compare the antibacterial effects of wasabi with AITC on the two pathogens. The results from this study may provide valuable information for the application of wasabi to control bacterial pathogens in foods, especially certain ready-to-eat foods as they become more popular and more frequently implicated in foodborne outbreaks. In addition, the results from this study may stimulate more research to search and evaluate other edible plants containing antimicrobial compounds for controlling foodborne pathogens.

## Materials and Methods

### Bacterial Cultures

Both *E. coli* O157:H7 (strain B0241) and *S*. *aureus* (strain B0031) were obtained from the culture collections in USDA Agricultural Research Service located at North Carolina State University. *E*. coli O157:H7 and *S*. *aureus* were originally isolated from bovine carcass and meat, respectively. The glycerol stocks of the two cultures were stored at -80°C until use.

### Fresh Culture Preparation

Tryptic Soy Broth (TSB) and Tryptic Soy Agar (TSA) were used to prepare fresh cultures. A frozen culture was streaked onto a TSA plate. The plate was then incubated at 37°C overnight. The broth culture was prepared by inoculating 10 ml of TSB with a colony from a TSA plate and incubated at 37°C overnight (12 to14 h).

### Wasabi

Commercially available wasabi powder was obtained from China. The power was packaged in small bags (10 g per bag) and ready for consumers to use in foods. After received, the wasabi powder from different bags was pooled, mixed, aliquoted, and then stored at -80°C until use.

### Moisture Content in Wasabi Powder

The moisture content in wasabi powder was measured by Intell-lab WPS 50SX moisture analyzer (DSC 50P, Data Support Company Inc., Panorama City, CA, USA) with the drying temperature at 100°C and drying time for 10 min.

### Chemicals

Allyl isothiocyanate (purity ≥ 94%), phenyl isothiocyanate (PITC, purity ≥ 98%), and ethanol were purchased from Thermo Fisher Scientific (USA). AITC and PITC were stored in the refrigerator until use. Since AITC is only slightly soluble in water, ethanol was used as a solvent to prepare AITC stock solutions at varying concentrations (0.01, 0.1, 1, 5, and 10%).

### Preparation of Wasabi Extract

**FIGURE 1:**
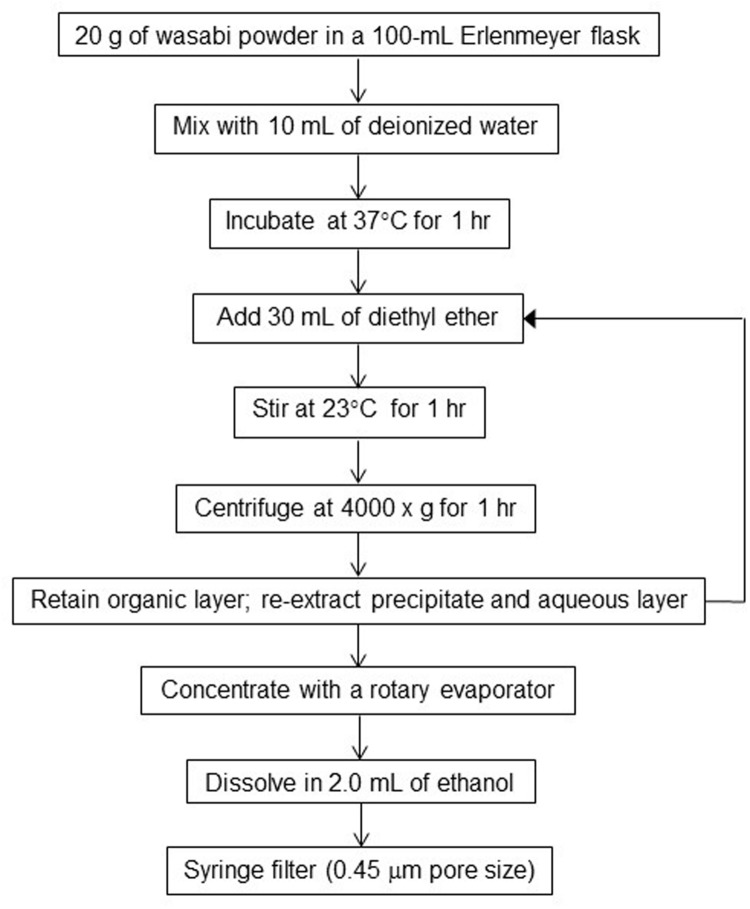
**Flowchart for preparation of wasabi extract**.

In order to determine the AITC concentration in wasabi, wasabi extract was prepared by using diethyl ether as a solvent according to the procedure described by Shin et al. (2004) with some modification. Briefly, 20 g of wasabi powder was mixed with 10 ml of deionized (DI) water and incubated at 37°C for 1 h (**Figure 1**). Thirty ml of diethyl ether was added to the mixture and stirred for 1 h at room temperature (23°C). The mixture was then centrifuged at 4000 × *g* for 1 h. The organic layer (diethyl ether phase) was transferred into a 500-ml round bottom flask. The remaining precipitate and aqueous layer were extracted with additional 30 ml of diethyl ether. The extraction process was repeated a total of three times (with a total of 90 mL of diethyl ether) to insure efficient extraction of AITC into the organic layer. The solvent in the organic layer was evaporated to dryness with a rotary evaporator. The dried sample (an organic film) was dissolved in 2 ml of ethanol. The resulting solution (wasabi extract) was filtered through a 13-mm nylon Fisherbrand syringe filter (0.45 μm pore size) into a GC-MS auto-sampler vial prior to chemical analysis.

### Quantitative Analysis of AITC in Wasabi

The AITC concentration in wasabi (powder) was determined according to the method described by Kumagai et al. (1994) and Shin et al. (2004) using PITC as an internal calibration standard with some modification. Briefly, wasabi extract (in ethanol) was subjected to the analysis by gas chromatography-mass spectroscopy (GC-MS). AITC concentration was measured by using split quantitative gas chromatography (Shimadzu GC-17) equipped with an SHRXI-5 ms capillary GC-column (30 m × 0.25 mm × 0.25 μm) and a mass spectrometer (Shimadzu QP2010-MS). The initial column oven temperature was held at 50°C for 1 min and increased to 200°C at a rate of 10°C/min. The final hold time was 3 min producing 19-min chromatograms. The temperature of the injection port and detector were kept at 220°C. The carrier gas was 53.5 kPa high purity helium (50 ml/min total flow, 1.0 ml/min column flow, 36.3 cm/s linear velocity, 3.0 ml/min purge flow). Initial experiments confirmed the absence of PITC in wasabi extract allowing its use as an internal standard for quantitative analysis of AITC. Qualitative identification of other compounds present in the chromatogram was achieved using the NIST08 mass spectrum library.

### Effect of Wasabi on the Growth of *E. coli* O157:H7 and *S. aureus*

The antibacterial activity of wasabi against *E*. *coli* O157:H7 or *S. aureus* was evaluated in 15-ml tubes. Each tube contained 10 ml of TSB, 0.5, 1, 2, or 4% of wasabi powder, and a fresh overnight culture (at the initial cell concentration around 10^5^ CFU/ml measured by the plate count method). A tube containing 10 ml of TSB and the same concentration of the culture without wasabi served as a control. In addition, another tube containing 10 ml of TSB and 100 mg of wasabi without added culture was also included in each set of experiment to see if any microflora was present in wasabi. All tubes were screw-capped and incubated at 37°C in a benchtop rocker incubator (SI-1400, Scientific Industries, Inc., Bohemia, NY, USA) with rocking speed at 14 cycles/min for 12 h. Samples were taken at 4-h intervals for 12 h. The same procedure was also used to evaluate the antibacterial activity of wasabi against *S. aureus*. The viable cell concentration in each sample was determined using the plate count method. Eosin Methylene Blue (EMB) agar plates and Mannitol Salt Agar (MSA) plates were used for enumerating *E*. *coli O157*:H7 and *S. aureus*, respectively. Each experiment was repeated at least two more times. MIC of wasabi was defined as the lowest wasabi concentration in TSB where no growth was observed after 8-h incubation at 37°C.

### Effect of AITC on the Growth of *E*. *coli* O157:H7 and *S. aureus*

The antibacterial activity of AITC was also evaluated in TSB at the same initial cell concentration (10^5^ CFU/ml) and under the same condition as described above except that one of the AITC stock solutions (100 μl), instead of wasabi powder, was added into each tube to reach the final AITC concentration of 1, 10, 100, 500, or 1000 μg/ml. Two types of control tubes were included in each set of experiment. The control tubes contained 10 ml of TSB and a bacterial culture supplemented with either 100 μl of water or 100 μl of ethanol. The control tube containing ethanol was used for evaluation of the possible inhibitory effect from ethanol (the solvent in AITC stock solution) on the bacterial growth. The same sampling and enumerating procedures described above were followed. Each experiment was repeated two more times. MIC of AITC was defined as the lowest AITC concentration in TSB where no growth was observed after 8-h incubation at 37°C.

### Statistical Analysis

One-way Analysis of variance (ANOVA) was conducted using Statistica for Windows (StatSoft, Tulsa, OK, USA). Tukey’s HSD test was used to compare the mean values of data for significant difference (*P* < 0.05).

## Results

### Moisture Content, AITC and other ITCs in Wasabi

**FIGURE 2:**
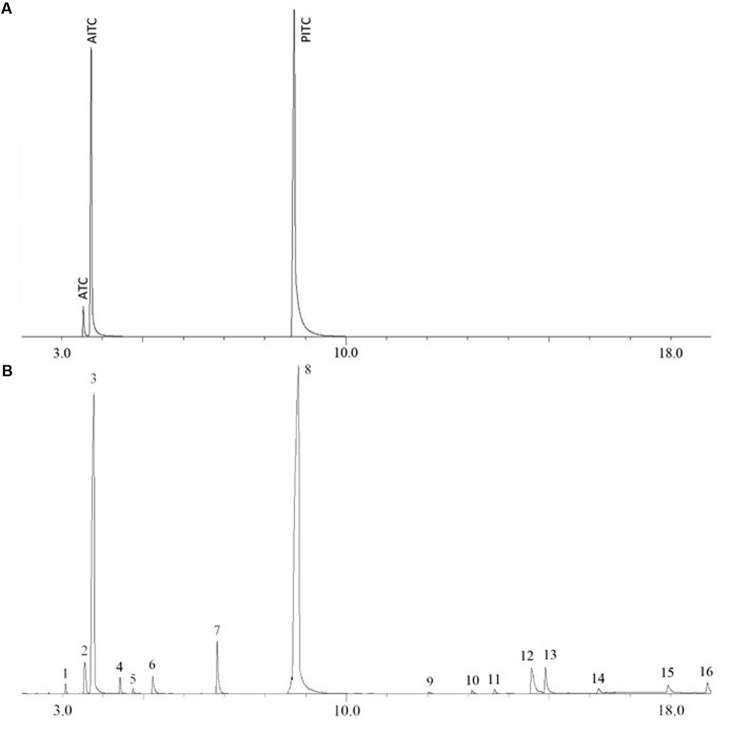
**Gas chromatograms of AITC and PITC standards (A) and wasabi extract with PITC as an internal standard (B)**.

The moisture content in the wasabi (powder) used in this study was measured to be 6.5% (wt/wt). AITC content in the wasabi was quantified by GC-MS with PITC as an internal standard. The retention times of AITC and PITC on gas chromatograms were around 3.73 and 8.74 min, respectively (**Figure 2A**). **Figure 2B** showed that the peak of AITC was several times higher than that of any other volatile compounds in wasabi. AITC concentration in the wasabi powder was measured to be 5.91 ± 0.59 mg/g. Thus, 0.5, 1, 2, and 4% of wasabi contained 29.6, 59, 118, and 236 μg/ml of AITC, respectively. Besides AITC, five other ITCs (isopropyl isothiocyanate, 1-isothiocyanatobutane, isobutyl isothiocyanate, 4-isothiocyanato-1-butene, and cyclopentyl isothiocyanate) and a few other compounds were also identified in wasabi (**Figure 2B** and **Table 1**). In addition, a small peak of allyl thiocyanate (ATC) to the left of AITC peak was observed from the standard consisting of AITC and PITC (**Figure 2A**) and from wasabi extract (**Figure 2B**), but not observed from the standard containing only PITC (data not shown).

**Table 1:** Volatile compounds in wasabi extract with an internal standard.

Peak #	Compound	Formula	M^+^	MS similarity index
1	Isopropyl isothiocyanate	(CH_3_)_2_CH-N = C = S	101	91%
2	Allyl thiocyanate (ATC)^a^	CH_2_ = CHCH_2_S-C≡N	99	90%
3	Allyl isothiocyanate (AITC)	CH_2_ = CHCH_2_-N = C = S	99	96%
4	1-Isothiocyanatobutane	CH_3_CH_2_CH_2_CH_2_-N = C = S	115	86%
5	Isobutyl isothiocyanate	(CH_3_)_2_CHCH_2_-N = C = S	115	92%
6	4-Isothiocyanato-1-butene	CH_2_= CHCH_2_CH_2_-N = C = S	113	88%
7	Cyclopentyl isothiocyanate	C_5_H_9_-N = C = S	127	76%
8	Phenyl isothiocyanate (PITC)^b^	C_6_H_5_-N = C = S	135	97%
9	Ethyl carbanilate	C_9_H_11_NO_2_	165	95%
10	Butylated hydroxytoluene	C_15_H_24_O	220	91%
11	sec-Butyl cyclopentyl sulfide	C_5_H_9_-S-C_4_H_9_	158	68%
12	N-Phenyl-N-(4-methyl-1,3,2-dioxaphospholane-2-yl) ethyl thiocarbamate	C_12_H_16_NO_3_PS	285	76%
13	Unknown compound	—	189	—
14	Undecylenic acid	C_11_H_20_O_2_	184	63%
15	Unknown compound	—	174	—
16	Palmitic acid	C_16_H_32_O_2_	256	96%

*^a^ATC was generated through thermal isomerization during GC analysis (see Section Discussion). ^b^PITC was an internal standard.*

### Effect of Wasabi on the Growth of *E*. *coli* O157:H7 and *S. aureus*

To check if any microflora was present in the wasabi (powder) used in this study, a tube containing 10 ml of TSB, 100 mg of wasabi powder, and no added bacteria was included in each set of experiments. After 12-h incubation, no viable cells were detected in this tube (data not shown), indicating that the microflora that might be originally present in the wasabi was inactivated during the preparation of the powder (such as drying and grounding) or a long term exposure to antibacterial agents present in wasabi. Therefore, the detected bacterial cells in inoculated tubes were solely from the inoculum.

**FIGURE 3:**
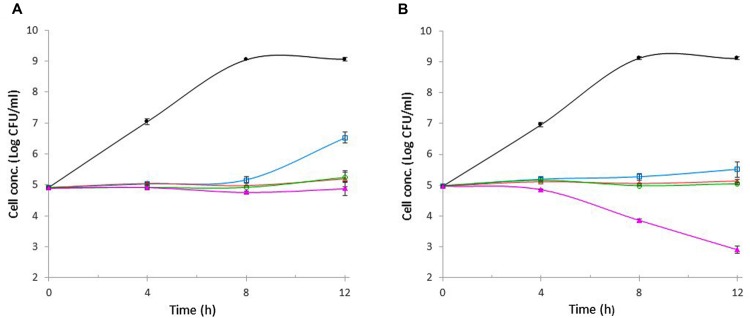
**Effect of wasabi on the growth of *E*. *coli* O157:H7 (A) and *S. aureus* (B)**. Wasabi concentration in each tube was 0%, 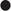; 0.5%, 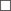; 1%, 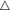; 2%, 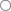; or 4%, 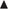. Each point represents the mean ± standard error based on three measurements.

The antibacterial activities of wasabi at varying concentrations (0.5, 1, 2, or 4%) against *E*. *coli* O157:H7 and *S. aureus* were evaluated separately at the initial cell concentration of 10^5^ CFU/ml over a 12-h period at 37°C. *E*. *coli* O157:H7 in the control tube (without wasabi) grew exponentially during the first 8 h, resulting in 4-log unit increase in cell concentration, and the final concentration reached 10^9^ CFU/ml which was significantly higher than that in any wasabi-containing tube (**Figure 3A**). In contrast, *E. coli* O157:H7 concentration increased slightly in the presence of 0.5% wasabi and remained unchanged in the presence of 1 or 2% wasabi during the 8-h incubation (**Figure 3**). As a result, bacterial concentrations in those tubes were about 4-log units lower than that in the control. After 12-h incubation, cell concentration in the tube containing 0.5% of wasabi increased 1.5-log units compared with initial cell concentration (**Figure 3**). But even so, the cell concentration was still 2.5-log units lower than that in the control. The cell concentrations in the tube with 1 or 2% of wasabi remained almost unchanged. A slight decrease in cell concentration was observed in the tube containing 4% of wasabi after 8-h incubation. The final cell concentrations in the tubes containing 1, 2, and 4% of wasabi were about 4-log units lower than that in the control. Similar growth profile was observed with *S. aureus* during the first 8-h incubation except that the exposure to 4% of wasabi caused *S. aureus* concentration to decrease exponentially after 4-h incubation resulting in more than 1-log reduction in cell concentration at hour 8 (**Figure 3B**). As the incubation continued, cell concentrations remained unchanged in all tubes except the tube containing 4% of wasabi in which *S. aureus* concentration continued to decrease exponentially and finally reached 10^3^ CFU/ml, which was 6-log units lower than that in the control (**Figure 3B**). These results indicated that the MICs of wasabi against *E*. *coli* O157:H7 and *S. aureus* were both 1% (equal to 10 mg/ml) and 4% of wasabi had significantly higher bactericidal activity against *S. aureus* than against *E*. *coli* O157:H7.

### Effect of AITC on the Growth of *E*. *coli* O157:H7 and *S. aureus*

**FIGURE 4:**
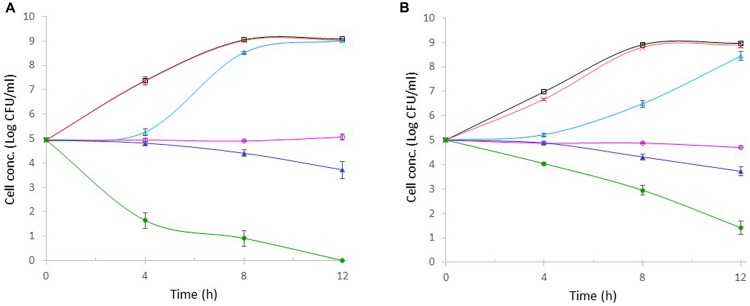
**Effect of AITC on the growth of *E*. *coli* O157:H7 (A) and *S. aureus* (B)**. AITC concentration in each tube was 0 μg/ml, 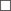; 1 μg/ml, ×; 10 μg/ml, 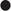; 100 μg/ml, 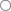; 500 μg/ml, 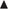; or 1000 μg/ml, 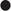. Each point represents the mean ± standard error based on three measurements.

Allyl isothiocyanate stock solution was prepared using ethanol as a solvent. The possible inhibitory effect of ethanol in AITC stock solution was evaluated in a control tube supplemented with 100 μl of ethanol. It was found that the bacterial growth (*E*. *coli* O157:H7 or *S. aureus*) in these control tubes containing ethanol (data not shown) was identical to that in the control tube containing no ethanol (**Figures 4A,B**), indicating that the inhibition (if any) from ethanol in 100 μl of AITC stock solution was negligible.

The antibacterial activities of AITC at varying concentrations (1, 10, 100, 500, or 1000 μg/ml) against *E*. *coli* O157:H7 and *S. aureus* were evaluated separately under the same condition. **Figure 4** showed that *E*. *coli* O157:H7 and *S. aureus* in the control tubes (without AITC) grew exponentially and entered stationary phase within 8-h incubation, reaching 10^9^ CFU/ml. In the presence of 1 μg/ml of AITC, the growth of either bacterium was not significantly different from that in the control. When AITC concentration was increased to 10 μg/ml, no bacterial growth was observed for almost 4 h. After 4 h, both bacteria started to grow. *E*. *coli* O157:H7 grew more rapidly and eventually entered stationary phase reaching 10^9^ CFU/ml (**Figure 4A**). *S. aureus* grew significantly slower than *E*. *coli* O157:H7, but it gradually entered stationary phase and its final concentration was only 0.5-log lower than 10^9^ CFU/ml in the control (**Figure 4B**). In the presence of 100 μg/ml of AITC, the concentrations of both bacteria remained unchanged during the 12-h incubation. As a result, bacterial concentration of *E*. *coli* O157:H7 or *S. aureus* was 4-log units lower than those in controls. These results indicated that the MIC of AITC against *E*. *coli* O157:H7 or *S. aureus* was higher than 10 μg/ml but lower than 100 μg/ml. Exposure to 500 μg/ml of AITC resulted in 0.6- and 1.2-log reduction in *E. coli* O157:H7 or *S. aureus* concentration within 8- and 12-h, respectively. Exposure to 1000 μg/ml AITC caused a rapid (3.7-log) reduction in *E. coli* O157:H7 concentration within 4 h (**Figure 4A**). The resulting *E. coli* O157:H7 concentration was 6-log units lower than that of the control. As the incubation continued, *E. coli* O157:H7 was undetectable, and the resulting cell concentration was 9-log units lower than the control. The cell death of *S. aureus* was significantly slower than *E. coli* O157:H7 during the first 4-h incubation and only 1-log reduction in cell concentration was observed (**Figure 4B**). Additional 1- and 3-log reductions in *S. aureus* concentration were achieved after 8- and 12-h incubations, respectively. The final *S. aureus* concentration was 8-log units lower than that in the control.

## Discussion

The moisture content in wasabi varies with cultivar, growing region, and the form of the products (leaves or stem; fresh or dry; cut or ground). The moisture content in fresh wasabi grown in New Zealand is around 81% (Sultana et al., 2003). The wasabi used in this study was dry powder containing 6.5% of moisture. It is commercially available and ready to use in foods. Wasabi contains fresh green flavor compounds, which are not found in horseradish (Ina et al., 1989; Masuda et al., 1996). The fresh “green” notes make wasabi more appealing to many consumers.

Allyl isothiocyanate is a volatile sulfur-containing compound naturally present in *Brassicaceae* plants and particularly abundant in wasabi and horseradish. AITC in wasabi is derived through the enzymatic hydrolysis of its precursor allyl glucosinolate when plant tissues are damaged by chewing, crushing, or grinding (McGregor et al., 1983). Like other ITCs, AITC is formed under neutral and alkaline conditions, but once formed it is more stable under acidic condition (Sultana et al., 2003). AITC content in wasabi varies with the cultivar, the growing region and conditions. It also varies with the parts of the same wasabi plant. It has been reported that AITC content in Japanese wasabi leaves, stems, and roots were 0.38, 0.41, and 1.18 mg/g, respectively, while AITC content in Korean wasabi leaves, stems, and roots were 0.32, 0.18, and 0.75 mg/g, respectively (Shin et al., 2004). Hasegawa et al. (1999) reported that AITC content in Japanese wasabi roots was 1.13 mg/g. The AITC content in the dry wasabi powder used in this study was 5.91 ± 0.59 mg/g, which was much higher than those in fresh Japanese or Korean wasabi leaves, stems, or roots.

The MICs of wasabi against *E. coli* O157:H7 and *S. aureus* were both 1% (equivalent to 10 mg/ml) which contained 59 μg/ml of AITC. This AITC concentration in 1% wasabi was within the MIC range of AITC (between 10 to 100 μg/ml) determined in this study. Since very little research was done to directly study the antibacterial activity of wasabi, no data on MIC of wasabi against *E. coli* O157:H7 and *S. aureus* can be found in the literature for comparison. Hasegawa et al. (1999) reported that the growth of *V. parahaemolyticus* AOTO-81 at 10^2^ CFU/ml in fatty tuna meat suspension was inhibited by 20 mg/ml of wasabi root which was grated, but not dried. Apparently, MIC of wasabi varies with bacteria, initial cell concentration, media, and, more importantly, wasabi (source and moisture content). Wasabi at 4% displayed significantly higher bactericidal activity against *S. aureus* than against *E. coli* O157:H7. It is unclear why there was such a difference in the bactericidal activity of wasabi between the two bacteria. More research is needed to better understand the mechanism of antibacterial activity of wasabi. The high antibacterial activities of wasabi shown in this study make wasabi more appealing to consumers who like wasabi. As people enjoy the natural color and the unique flavor of wasabi, they also obtain the health benefit of consuming wasabi which offers an advantage to safeguard foods against pathogens at the point of ingestion. A study is underway to evaluate the effectiveness of wasabi against other strains of *E. coli* O157:H7 and *S. aureus* as well as other major foodborne pathogens including *L. monocytogenes* and *S. typhimurium* in ready-to-eat foods. As the health benefits of consuming wasabi become better known, the production and consumption of this interesting edible plant are expected to be increased.

Chemical analysis showed that AITC is the major isothiocyanate in wasabi, and other ITCs are present at very low concentrations. These results were in agreement with those in the literature (Kumagai et al., 1994; Hasegawa et al., 1999; Shin et al., 2004). Sultana et al. (2003) reported that AITC counts for 94% of the total ITCs in wasabi. Our study identified five other ITCs in wasabi. Four of them (isopropyl ITC, 1-isothiocyanatobutane, isobutyl ITC, 4-isothiocyanato-1-butene, and 1-isothocyanatobutane) were also identified in wasabi by other researchers (Kumagai et al., 1994; Masuda et al., 1996; Sultana et al., 2003; Shin et al., 2004). Several studies showed that these ITCs had inhibitory effects on certain foodborne pathogens including *E. coli* O157:H7 and *S. aureus* (Lin et al., 2000a; Shin et al., 2004; Dias et al., 2014; Dufour et al., 2015). Therefore, they contributed to the total antibacterial activity of wasabi even though their concentrations were very low. It has been reported that ITC compounds with shorter carbon chains have stronger antibacterial activity due to higher efficiency and stability in linkage with bacteria structures (Wilson et al., 2013).

The fact that ATC peak was present in the standard consisting of AITC and PITC and in wasabi extract but absent in PITC-only standard suggested that ATC may not be naturally present in wasabi. It was suspected that ATC might be produced from the thermal isomerization of AITC during GC analysis. Chen and Ho (1998) demonstrated that AITC is unstable at high temperature and it can be decomposed to other compounds under cooking condition. They reported that ATC can also be thermally generated from AITC when pure AITC was introduced into the GC through the heated injection port. Kumagai et al. (1994) showed the same small peak to the left of AITC peak in all four chromatograms of wasabi extracts. However, they seemed to mistakenly identify it as hexanol because hexanol is neither naturally occurring in wasabi and nor a thermal degradation product of AITC. The same small peak to the left of AITC peak was also observed in every chromatogram reported by Shin et al. (2004), but they did not attempt to label and identify it.

The MICs of AITC against *E. coli* O157:H7 and *S. aureus* were both between 10 and 100 μg/ml. Further research is needed to determine the exact MIC values against the two pathogens. Dufour et al. (2012) reported that the MIC of AITC against a panel of 24 *Campylobacter jejuni* isolates was in the range of 50–200 μg/ml. Hasegawa et al. (1999) reported that AITC at 101.7 μg/ml effectively inhibited four of the *V. parahaemolyticus* strains while AITC at 50.9 μg/ml inhibited only one *Vibrio* strain in fatty tuna meat suspension. Various methods have been used to determine MIC of AITC, such as agar dilution method, paper disk agar diffusion method, viable colony counting, and time-to-kill assay. MIC values can be greatly influenced by the method used. It is difficult to compare published MIC values of AITC because they were not measured under well standardized conditions (Dufour et al., 2015). There is no general rule concerning the efficacy of AITC toward various bacteria.

Allyl isothiocyanate at 500 μg/ml showed similar (not significantly different) bactericidal activity against both *E*. *coli* O157:H7 and *S. aureus* resulting in 1.2-log reduction in cell concentration. But higher AITC concentration (1000 μg/ml in this study) killed *E*. *coli* O157:H7 significantly faster than *S. aureus* resulting in 3.7-log reduction in *E*. *coli* O157:H7 concentration and only 1-log reduction in *S. aureus* concentration within 4 h. Similar observation was also made by Shin et al. (2004) who reported that the minimum bactericidal concentrations of AITC against *E*. *coli* O157:H7 ATCC 43889 and *S. aureus* ATCC 25923 were 670 and 2670 μg/ml, respectively. Lin et al. (2000b) reported that *Salmonella Montevideo* and *E. coli* O157:H7 were more sensitive to AITC than *Listeria monocytogenes*. Such a difference is likely due to the difference in cell wall structure between Gram-positive and Gram-negative bacteria. The thicker cell wall in Gram-positive bacteria may better prevent or slow down the diffusion of AITC into the cells, making cells less sensitive to AITC. Our data showed that AITC at 500 μg/ml did not cause significant difference in its bactericidal activity against *E*. *coli* O157:H7 and *S. aureus*. But AITC at 1000 μg/ml resulted in significantly more rapid reduction in *E*. *coli* population. More research is needed to study other Gram-positive and Gram-negative bacteria at various concentrations of AITC. It has been reported that AITC can cause membrane damage, making it more permeable, thereby resulting in leakage of cellular metabolites, similar to the effect of polymyxin B (Lin et al., 2000b). AITC can also inhibit thioredoxin reductase and acetate kinase in *E. coli* O157:H7 (Luciano and Holley, 2009). However, the exact antibacterial mechanisms of AITC and other isothiocyanates are not well understood (Dufour et al., 2015). It is worth to note than although AITC is the major antibacterial compound present in wasabi, natural wasabi is still more appealing than AITC or wasabi extract to consumers for antibacterial property.

## Conclusion

The study showed that wasabi as well as AITC at low concentrations was bacteriostatic and bactericidal at high concentrations against *E. coli* O157:H7 and *S. aureus*. Wasabi has a high potential to effectively control *E. coli* O157:H7 and *S. aureus* and possible other foodborne pathogens in foods. The antibacterial property along with its natural green color, unique flavor, and advantage to safeguard foods at the point of ingestion makes wasabi a promising natural edible antibacterial plant. The results from this study may be of significant interest to the food industry as they develop new and safe foods. These results may also stimulate more research to evaluate the antibacterial effect of wasabi against other foodborne pathogens and to explore other edible plants for their antimicrobial properties. To our knowledge, this is the first report on the antibacterial activity of wasabi in its natural form of consumption against *E. coli* O157:H7 and *S. aureus*.

## Author Contributions

ZL designed experiments, conducted all microbiology experiments, analyzed data, prepared the manuscript. CD prepared wasabi extract, conducted chemical analysis, reviewed the manuscript. MC and KC conducted microbiology experiments. BP and MG were involved in wasabi extract prep and chemical analysis.

## Conflict of Interest Statement

The authors declare that the research was conducted in the absence of any commercial or financial relationships that could be construed as a potential conflict of interest.
